# Intimate partner violence against women on the Colombia Ecuador border: a mixed-methods analysis of the liminal migrant experience

**DOI:** 10.1186/s13031-021-00351-y

**Published:** 2021-04-08

**Authors:** Colleen Keating, Sarah Treves-Kagan, Ana Maria Buller

**Affiliations:** 1 London School of Hygiene and Tropical Medicine, Keppel St, London, England; 2 Department of Health Behaviour, Gillings School of Global Public Health, University of North Carolina at Chapel Hill, Chapel Hill, USA; 3 Department of Global Health and Development, London School of Hygiene and Tropical Medicine, 15-17 Tavistock Place, London, WC1H 9SH England

**Keywords:** Displaced women, Intimate partner violence, Latin America, Liminality

## Abstract

**Background:**

Intimate partner violence (IPV) has serious long-term health and psychological consequences and is highly prevalent in Latin America and among displaced populations. Liminality - the ambiguous in-between state of individuals completing a migratory journey - represents a state of legal, economic, and physical insecurity. Through the framework of liminality, this analysis seeks to understand the unique challenges faced by displaced Colombian women in Ecuador including their experience of IPV.

**Methods:**

We performed a secondary analysis of 15 in-depth interviews and 319 longitudinal surveys, conducted on the border of Ecuador and Colombia, following a sequential explanatory mixed-methods design. We analysed interviews thematically and mapped the main themes onto complementary quantitative variables. We conducted logistic regression with identified risk and protective factors (measured at time 1) and recent IPV (measured at time 2), controlling for demographic characteristics and IPV at time 1.

**Results:**

Our mixed-methods analysis revealed four main mechanisms by which displacement influenced the social and economic realities of Colombian women years after crossing the border, compounding their risk of IPV and limiting their ability to escape it. Lack of legal residence and documentation, violence experienced along life course and migratory continuums which increased their risk for later revictimisation, social isolation including loss of support networks and restricted mobility and lastly, financial stress.

**Conclusions:**

This research highlights the critical importance of supporting the economic and social integration of migrants and refugees in host communities, as well as the need to carefully consider migration-related vulnerabilities in IPV prevention and response interventions. As the regional refugee crisis grows, policy makers must consider how the long-term marginalisation of refugee women contributes to their victimisation. This research also supports the idea of incorporating gender synchronised, transformative IPV prevention and response programmes into migration-related and poverty alleviation international development efforts.

## Background

Intimate partner violence (IPV) refers to psychological, physical, or sexual violence by current or former partners and it constitutes a serious but preventable public health issue [1]. It impacts over 40% of women in the Andean region of Latin America [2] and results in significant health, social, and economic consequences. Displaced women and women living in conflict-affected communities face exacerbated vulnerabilities and risk factors for IPV, regardless of immigration status [3–5]. Trauma, poverty, changing gender roles, and the general stress of violence and displacement bolster existing levels of IPV and prevent women from accessing help [5–7]. In some settings, exposure to political violence has been associated with increased risk of IPV victimisation for women and for increased risk of IPV perpetration for men [8, 9].

Liminality describes a state of transition or passage [10, 11]. The term has been appropriately used to describe the ambiguous in-between state of individuals completing migratory journeys, and borderlands themselves characterised by their social, economic, legal, and physical insecurity [12]. Hence, liminality provides a theoretical framework to understand the peculiarities of the experiences of refugee women around the world in border regions. Menjívar, for instance, describes Salvadoran and Guatemalan immigrants in the United States (US) as experiencing an ambiguous documentation status that affects migrant’s social networks and sense of belonging [13]. McGuire and Georges, and Stephen on the other hand, describe the US-Mexico border as a physically and legally liminal place for indigenous Oaxacan women migrating to the US [14] and where IPV must be understood within the context of structural violence committed by the state against undocumented migrants [15]. Liminality has also been found to exacerbate the experience of IPV for Polish women living in the British borderlands permeating their political subjectivities, and their ability to achieve safety and protection from abuse [16].

Colombia’s civil war between the government and non-state armed groups has displaced over five million Colombians. Most migration occurred from rural areas to urban centres within the country, but large numbers of Colombians migrated internationally. An estimated 250,000 Colombian refugees lived in Ecuador at the time of this study [17]. Displaced Colombians are disproportionately poor and illiterate and are often racial minorities [18]. Refugees face high levels of joblessness, as few employment opportunities exist for those who have no skills beyond subsistence farming [17]. Qualitative research conducted in Colombia found that physical and sexual violence were equally prevalent among displaced women and women who remained in conflict-affected communities, but noted higher reports of “opportunistic violence” including abduction, rape, and trafficking among displaced women [7]. For migrant Colombian women living in the border regions in Ecuador, as for other migrant women around the world [19, 20], liminality compounds their risk of IPV by denying them access to protective legal and social networks, increasing their odds of experiencing harmful events as they navigate border-crossings and resettle into foreign communities.

In order to reduce IPV in Ecuador and in contexts with similar migration patterns from neighbouring countries, it is crucial to understand the migrant experience and incorporate this learning into prevention and intervention efforts. Analysing data from the Ecuador-Colombian border during a crisis through the lens of liminality allows us to understand how the manifestation of violence changes across the migratory journey, and how the social, economic, and legal marginalisation of migrants allows IPV to persist or grow. Using the triangulation of quantitative and qualitative data and the framework of liminality, this paper aims to contribute to the current literature by shedding light into the unique IPV experience of displaced Colombian women living in the Ecuador border.

## Methods

We conducted a secondary analysis of a mixed methods sequential explanatory study, triangulating data from longitudinal surveys and in-depth interviews collected from Colombian refugee women living in Ecuador. This study draws on data from a cluster randomised controlled trial of a World Food Programme (WFP) Cash, Voucher, and Food Transfer intervention conducted by the International Food Policy Research Institute (IFPRI) in northern Ecuador in 2011. Longitudinal survey data was collected to assess the study’s impact, which improved food insecurity, IPV, and social cohesion outcomes [21, 22]. Qualitative data was then collected to examine the pathways through which the intervention served to reduce IPV [23], using the IPV quantitative results to guide the sampling. For the purpose of this study, we conducted a secondary analysis of a sub-sample of the qualitative (15/48) and quantitative (319/ 2122) data which focuses on the experience of IPV for Colombian women independently of the impact of the intervention.

### Study settings

**Fig. 1:**
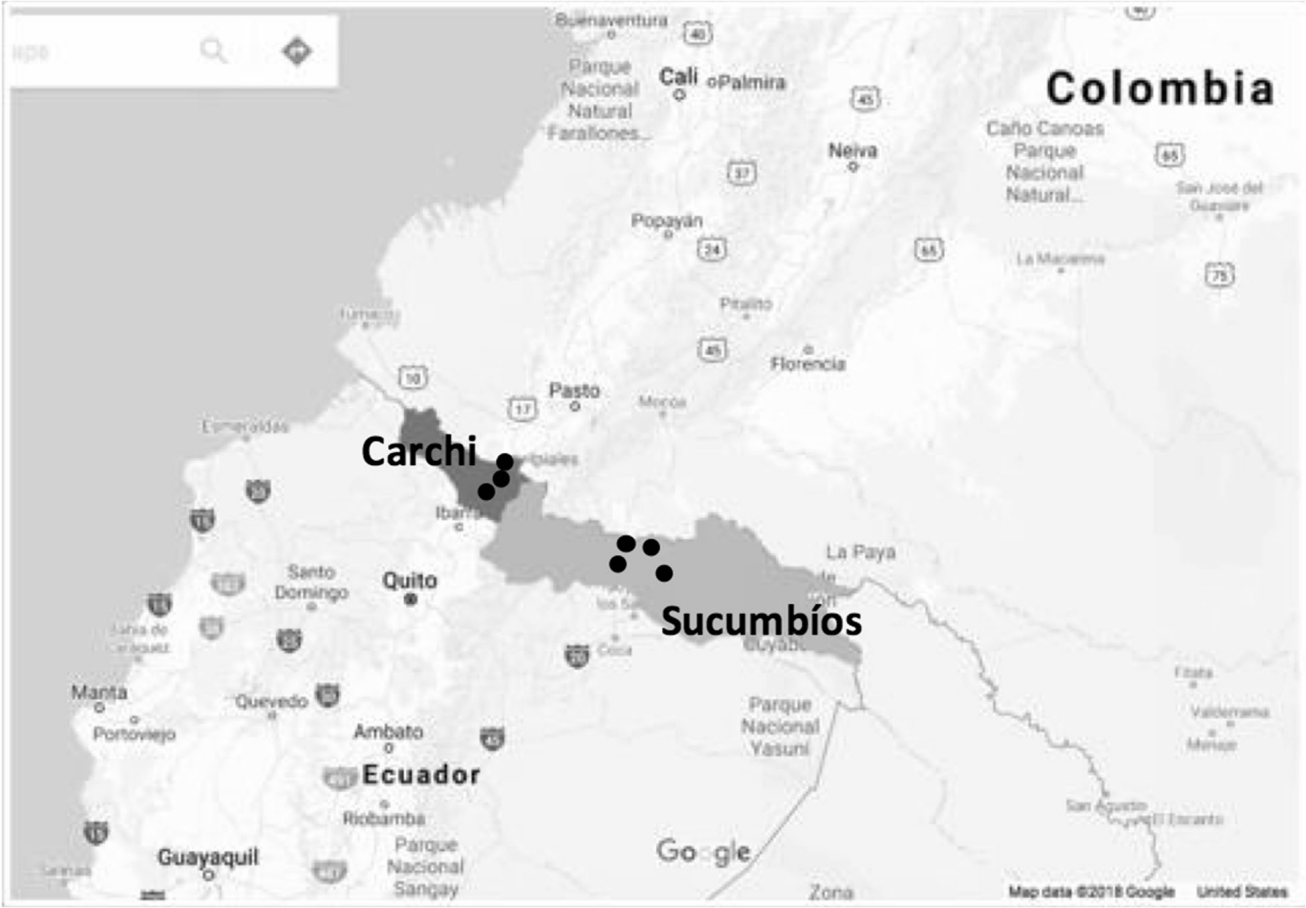
Map of Carchi and Sucumbios (study sites)

The study was conducted in the provinces of Carchi and Sucumbíos, a border region characterised by violence, and smuggling. Tulcán, the capital city of the Andean province Carchi, is located less than 10 kilometres from the Colombian border (Fig. 1). As Ecuador’s final stop on the Pan-American highway, the Rumichaca Bridge—which is less than 5 miles from Tulcán—is an important commercial link between the two countries. Nueva Loja is the capital city of Sucumbíos, the north eastern Ecuadorian province in the Amazon rainforest bordering Colombia. The city has become an industrial hub for the oil industry, and also serves as a critical gateway connecting drug cartels to US and European export markets. In a report to the Department of National Security & Strategy, US Army colonels described Nueva Loja as an “ungoverned territory” and a “lawless area” that serves as a pipeline into Ecuador for cocaine, weapons, humans, chemicals, and millions of US dollars [24].

At the time of the study, approximately half of the 250,000 Colombian refugees in Ecuador resided in the northern border region [17]. A Refugee Council report described the Colombia-Ecuador border as “porous,” referring to the frequent interstate movement of goods and persons. In this context, individuals often migrate seasonally for agricultural work, or more frequently to participate in the smuggling economy [17]. Part of this cyclical movement includes forced trafficking of women and children. As guerrilla activity increased in the region in the decade leading up to the study period, the permeable border allowed the Colombian conflict to spread over into Ecuador [24], and the security that many refugees sought became increasingly in jeopardy.

### Participants

Seven urban centres within Carchi and Sucumbíos with a high concentration of poverty and refugees were included in the study [23]. Within those cities, neighbourhoods with a large percentage of Colombian and low-income households were included in the sample. Neighbourhoods were then enumerated and 20–27 low-income households were randomly selected for participation; 2357 households were surveyed at time 1 and 2122 households were available for follow-up approximately seven to 8 months later. In total, 319 women self-identified as Colombian, were partnered at both time points, answered IPV questions at both time points, and were either the spouse of the head of household, or head of the household.

The original qualitative sample of 48 in-depth interviews was determined using a nested approach in which chosen participants represented a subset of the quantitative sample [25]. The decision on who to include was guided by the quantitative results around IPV. All of the women included in the interviews answered questions on IPV in the quantitative study at both time points, and all of them disclosed violence at time 1 [23]. Given the focus of this sub-study on IPV and migration, of the original 48 interviews, this analysis focused on the 15 women who self-identified as Colombian.

### Data collection

Quantitative data were collected in March–April 2011 and approximately 7 months later (October–November 2011) [21]. Paper surveys were administered by trained field staff, and verbal consent was collected. In-depth interviews were collected approximately 2 years later in Tulcán in Carchi and Nueva Loja in Sucumbíos by two women from Quito with previous interviewing experience in these geographical areas. Interviews were recorded and transcribed in Spanish. For more information about the methodology of the original study see Buller et al. [23].

### Analysis

We examined both quantitative and qualitative data to achieve complementarity between findings emerging from each method and to identify areas where results converged, diverged, or added insight to one another in relation to the topics of displacement and IPV. First, we explored qualitative data to identify key themes, and then mapped themes onto corresponding quantitative variables, which were analysed and then compared.

Qualitative data was analysed in Spanish using NVivo 12. Previous research was used to identify potential themes and inform the preliminary coding framework. The first author (who is proficient in Spanish) then read all interviews to become familiar with the structure and content of the interviews, and then re-read and summarised interviews to identify overarching themes, including emergent themes that had not been originally included in the preliminary coding framework such as social isolation and economic insecurity. Excerpts were translated from Spanish to English in the last phase of the analysis to include quotes in the manuscript.

The main themes arising from this analysis were then mapped onto available quantitative survey variables. The outcome variables were measured at follow-up and assessed women’s experience of emotional, physical and/or sexual IPV in the last 6 months (corresponding to the time between data collection points) using the World Health Organisation (WHO) Violence Against Women Instrument [26, 27]. Two dichotomous variables were constructed from 13 measures of emotional, physical, or sexual IPV: having experienced emotional IPV in the last six months (yes/no) and having experienced physical and/or sexual violence in the last six months (yes/no). Independent variables included forced migration; migration-related adversity; household wealth, value of women-owned assets, asset ratio between men and women partners, earning difference between partners, women labour force participation, and measures of social isolation, freedom of mobility, and trust in neighbours and community (see Table 2).

We assessed bivariate associations with IPV and the independent variables, and conducted logistic regression models. The main dependent variable was recent IPV at time 2 while independent variables were measured at time 1. Logistic regression models controlled for demographic variables (province, gender of head of household, women age and nationality, and men’s participation in the labour force), intervention status, and IPV at time 1. Maximum likelihood parameter estimates were generated with standard errors robust to non-normality and non-independence of observations. Adjusted odds ratios (AOR) and 95% confidence intervals (CI) are reported.

### Ethical issues

Ethics approval for the data collection was obtained from the International Food Policy Research Institute (IFPRI), LSHTM, and Centro de Estudios de Población y Desarrollo Social (CEPAR). For the qualitative secondary analysis, ethics approval was granted by LSHTM; University of North Carolina at Chapel Hill approved secondary analysis of quantitative data. Verbal consent was collected for both qualitative and quantitative participants. Field staff were trained by London School of Hygiene and Tropical Medicine (LSHTM) staff in accordance with the WHO ethical guidelines for conducting research on IPV including modules on ethical recruitment, consent, and data collection procedures; ensuring participant confidentiality, safety, and privacy; and managing data to maintain confidentiality. Specific procedures were followed during data collection to ensure the safety of the participants. For example, field survey staff only asked IPV questions if there were no other family members present, including her partner. In addition, anonymised referral information was offered to the women after completing the quantitative IPV module, regardless of whether the women had disclosed IPV or not.

Qualitative interviewers were local Ecuadorian women with experience interviewing vulnerable populations and trained in accordance with WHO guidelines on IPV research. Given the content of some of the interviewers, the fieldwork supervisor debriefed with interviewers every other evening to monitor for signs of emotional distress. Women who disclosed violence were provided with information about local support services, and a connection was established with a local refugee support centre prior to data collection to establish a referral system. Transcripts were anonymised and audio files stored under encryption. Due to the small sample of interviews, all names have been changed to pseudonyms and all identifying information has been omitted.

## Results

Tables 1 and 2 describe the demographic characteristics of the qualitative and quantitative samples. In the qualitative sample, the average participant age was 38.27 years; the mean number of years lived in Ecuador was 15; and 46% reported participating in the informal labour market. In the quantitative sample, the mean age was 32.96, 77.1% of women reported primary education or less as their highest educational achievement, and only 2.8% of women reported being the head of household. As reported in Table 3, 29 and 15% of Colombian women reported emotional IPV and physical and/or sexual violence, respectively.

**Table 1:** Qualitative sample (*n* = 15) characteristics

Name	Location	Age	Years in Ecuador	Employment*	Housing**
Maria	Tulcán	30	6	Unemployed	Rents
Sonia	Tulcán	28	9	Informal labour	Rents
Anita	Tulcán	47	10	Unemployed	Rents
Rosa	Tulcán	25	12	Informal labour	Rents
Isabel	Tulcán	47	12	Unemployed	Rents
Daniela	Tulcán	30	12	Unemployed	Rents
Alegria	Tulcán	45	14	Informal labour	Rents
Karla	Tulcán	52	26	Informal labour	Family owns
Renata	Tulcán	42	30	Informal labour	Rents
Emilia	Tulcán	44	40	Unemployed	Rents
Carolina	Nueva Loja	40	3	Informal labour	Rents
Esther	Nueva Loja	40	9	Unemployed	Lives with in-laws
Paula	Nueva Loja	33	11	Unemployed	Rents
Claudia	Nueva Loja	31	11	Unemployed	Family owns
Valeria	Nueva Loja	40	20	Informal labour	Family owns

*Informal labour refers to laundry service, selling fruit, working in a bar, or cleaning houses

**Where a house is family owned, the deeds to the houses were in the name of a husband or daughter

**Table 2:** Quantitative sample (*n* = 319) characteristics at time 1

	*n*	%
Emotional IPV in the last six months (time 2)	85	26.65
Physical and/or sexual IPV in the last six months (time 2)	46	14.42
**Demographics**		
Age (Mean years)		32.96
Highest Education Level		
Primary or less	246	77.10
Secondary or higher	73	22.90
Female headed households	9	2.82
Province		
Carchi	124	38.90
Sucumbíos	195	61.10
**Previous Trauma**		
Forced Migration	129	40.4
Migration-related adversity (Experienced by anyone in household)		
Verbally threatened	74	23.2
Verbally insulted but not threatened	80	25.1
Threatened with a knife, gun or other type of weapon	37	11.6
Attacked with a knife, gun or other type of weapon	23	7.2
Wounded or physically attached in some other way	23	7.2
Kidnapped	10	3.13
Forced to perform manual or some other kind of labour	19	5.96
Extorted for money or other goods	14	4.4
Stolen or damaged property	49	15.4
Forced to join a military group	21	6.58
Sexual aggression (rape, verbal or physical assault)	16	5
**Social Isolation**		
Family in area when moved here	134	42
Allowed to leave city without permission	32	10
I can count on my neighbour to watch after my house	111	34.8
I feel part of the community	40	12.5
I feel alone	198	62.1
Perceived discrimination because of race/ethnicity in last six months	19	5.96
Perceived discrimination because of nationality in last six months	116	36.4
**Economic Instability**		
Rent	266	83.4
Food Consumption Score (1)		56
Household wealth (2)		−0.08
Value of female assets (USD)		51.1
Asset ratio between male and female partner (3)		33.7
Female earns same or more as their partner	48	15.05
Female lost employment (4)	45	14.11
Female gained employment (4)	48	15.05

1. Calculated by summing the number of days eight different food groups were consumed by a household, multiplying these by weighted frequencies, and summing across food groups

2. Constructed as recommended by the Demographic Health Survey using principal component analysis of source of drinking water, type of toilet, sharing of toilet facilities, material of principal floor, walls, roof, cooking fuel, and household services and possessions, such as electricity, TV, radio, types of vehicles, agricultural land size owned, and type and number of animals owned

3. Assets include: land, large and small animals, agricultural tools, household appliances, computers, cell phones, bikes and vehicles

4. Participants were categorised as participating in the labour force if they responded yes to any of the following questions: In the last six months have you 1) farmed your own land; 2) worked in an agricultural position for pay; 3) worked in a non-agricultural position for pay; or 4) worked on non-agricultural activities, on their own, like a small business

**Table 3:** Percent of reporting recent IPV at time 1 and time 2*

	Time 1		Time 2	
	*n*	%	n	%
**Any emotional IPV in the last six months**	75	23.51	85	26.65
Accused of infidelity	34	10.66	36	11.29
Limited contact with friends or family	28	8.78	29	9.09
Humiliated or insulted you	50	15.67	56	17.55
Threatened to leave you	39	12.23	44	13.79
Threatened to take away your children	18	5.64	29	9.09
**Any physical and/or sexual IPV in the last six months**	51	15.99	46	14.42
Pushed, shook or threw something at you	41	12.85	30	9.40
Slapped you or twisted your arm	25	7.84	23	7.21
Hit you with a fist or something that could hurt you	22	6.90	20	6.27
Kicked or dragged you	10	3.13	12	3.76
Tried to strangle or burn you	4	1.25	9	2.82
Attacked you with a knife, gun or other type of weapon	1	0.31	3	0.94
Threatened you with a knife, gun or other type of weapon	3	0.94	1	0.31
Used physical force to force you to have sex	8	2.51	9	2.82
Forced you to perform sexual acts that you did not want to do	5	1.57	8	2.51

*Missing values not inputted

Below we explore the four main themes identified through the mixed methods analysis, which include lack of legal residence and documentation, violence experienced along life course and migratory continuums, social isolation including loss of support networks and restricted mobility, and financial stress. Table 4 shows the quantitative associations within each of the four key themes and Table 5 compares quantitative and qualitative findings.

**Table 4:** Logistic regression model of time 1 predictors of recent intimate partner violence at time 2 (*n* = 319)

	Physical and/or Sexual IPV				Emotional IPV			
	OR	95% CI		*P*-value	OR	95% CI		*P*-value
**Previous Trauma**								
Forced Migration (0 = No, 1 = Yes)	**5.06**	**2.03**	**12.59**	**0.00**	1.57	0.86	2.87	0.15
Migration-related adversity (Experienced by anyone in household) (0 = No, 1 = Yes)					0.63	0.24	1.65	0.35
Verbally threatened	**4.37**	**1.65**	**11.60**	**0.00**				
Verbally insulted but not threatened	0.47	0.17	1.28	0.14	1.28	0.65	2.51	0.48
Threatened with a knife, gun or other type of weapon	1.15	0.25	5.18	0.86	0.76	0.25	2.32	0.63
Attacked with a knife, gun or other type of weapon	1.46	0.34	6.20	0.61	1.83	0.72	4.69	0.21
Wounded or physically attached in some other way	**4.62**	**1.25**	**17.06**	**0.02**	0.51	0.14	1.78	0.29
Kidnapped	1.84	0.12	28.10	0.66	0.25	0.04	1.76	0.17
Forced to perform manual or some other kind of labour	0.70	0.06	7.93	0.78	2.29	0.46	11.37	0.31
Extorted for money or other goods	1.62	0.16	16.18	0.68	1.78	0.35	8.95	0.49
Stolen or damaged property	1.52	0.48	4.80	0.48	2.23	0.89	5.58	0.09
Forced to join a military group	**0.06**	**0.01**	**0.57**	**0.01**	0.73	0.14	3.70	0.70
Sexual aggression (rape, verbal or physical assault)	0.34	0.04	3.24	0.35	0.51	0.13	1.90	0.31
Emotional IPV at Time 1	–	–	–	–	**10.64**	**5.02**	**22.58**	**0.00**
Physical/and or Sexual IPV at Time 1	**14.48**	**5.81**	**36.10**	**0.00**	–	–	–	–
**Social Isolation**								
Family in area when moved here (0 = No, 1 = Yes)	0.66	0.26	1.64	0.37	**0.50**	**0.26**	**0.96**	**0.04**
Allowed to leave city without permission (0 = No, 1 = Yes)	0.87	0.27	2.82	0.82	1.01	0.35	2.91	0.99
Husband limits contact with family (0 = No, 1 = Yes)	**4.90**	**1.96**	**12.24**	**0.00**	–	–	–	–
I can count on my neighbour to watch after my house (0 = Disagree; 1 = Agree)	0.71	0.22	2.24	0.56	1.40	0.75	2.62	0.29
I feel part of the community (0 = Disagree; 1 = Agree)	0.48	0.15	1.56	0.22	0.87	0.29	2.60	0.80
I feel alone (0 = Disagree; 1 = Agree)	0.63	0.28	1.40	0.26	1.22	0.65	2.29	0.54
Perceived discrimination because of race/ethnicity in last six months (0 = No, 1 = Yes)	0.79	0.13	4.75	0.80	1.87	0.52	6.74	0.34
Perceived discrimination because of nationality in last six months (0 = No, 1 = Yes)	1.10	0.44	2.79	0.84	1.16	0.68	1.99	0.59
**Economic Instability**								
Rent (0 = No, 1 = Yes)	**4.42**	**1.95**	**10.05**	**0.00**	0.52	0.24	1.16	0.11
Food Consumption Score (1)	0.99	0.97	1.02	0.45	1.01	0.99	1.03	0.51
Household wealth (2)	0.92	0.55	1.53	0.74	0.91	0.63	1.31	0.61
Value of female assets (USD)	**0.998**	**0.995**	**1.000**	**0.02**	1.00	1.00	1.00	0.68
Asset ratio between male and female partner	2.20	0.61	7.97	0.23	1.75	0.68	4.54	0.25
Earning difference between partners (0 = Female earns less; 1 = Female earns same or more)	0.63	0.17	2.37	0.49	1.05	0.41	2.70	0.92
Female leaving labour force (4)	0.57	0.20	1.68	0.31	1.07	0.50	2.29	0.86
Female entering labour force (4)	3.09	0.94	10.13	0.06	1.46	0.73	2.90	0.29
Intercept	0.03	0.00	2.51	0.12	0.98	0.95	1.01	0.21

Model Notes: Adjusted for female age and education, province, treatment assignment, and baseline IPV; standard errors robust to non-normality and non-independence of observations; missings not imputed

1. Calculated by summing the number of days eight different food groups were consumed by a household, multiplying these by weighted frequencies, and summing across food groups

2. Constructed as recommended by the Demographic Health Survey using principal component analysis of source of drinking water, type of toilet, sharing of toilet facilities, material of principal floor, walls, roof, cooking fuel, and household services and possessions, such as electricity, TV, radio, types of vehicles, agricultural land size owned, and type and number of animals owned

3. Assets include: land, large and small animals, agricultural tools, household appliances, computers, cell phones, bikes and vehicles

4. Participants were categorised as participating in the labour force if they responded yes to any of the following questions: In the last six months have you 1) farmed your own land; 2) worked in an agricultural position for pay; 3) worked in a non-agricultural position for pay; or 4) worked on non-agricultural activities, on their own, like a small business

**Table 5:** Comparison of Quantitative and Qualitative Results

	Quantitative summary	Qualitative summary
**Living on the border and legal status**		
Legal status	No quantitative data was collected on legal status	Though interviews did not explicitly ask about legal status, many of the women disclosed their lack of formal documentation. Without legal residency in Ecuador, Colombian women were unable to travel freely across the border, own property, and secure formal employment. They conveyed stress to interviewers about the insecurity of their status in Ecuador and as Colombians felt discriminated against by their new communities.
History of abuse	For both emotional IPV and physical and/or sexual IPV, IPV at time 1 is highly associated with IPV at time 2 (*p* < 0.001). No quantitative data was collected on childhood maltreatment.	Two women described physical and sexual abuse as children, and another described IPV in a previous marriage. All but three of the women described a fairly continuous history of IPV with their current partners.
Guerrilla violence	Over 40% of Colombian woman who migrated to the area in the last 20 years, did so because of fear of violence, political reasons, food insecurity, property destruction or other reasons not related to marriage or looking for work. This was associated with recent P/S IPV (AOR: 5.06; 95% CI: 2.03, 12.59; *p* < 0.001). Having someone in the household verbally threatened (AOR: 4.37; 95% CI: 1.65, 11.60; *p* < 0.001) or wounded/physically attacked (AOR: 4.62; 1.25, 17.06; *p* < 0.05) since migrating was also associated with P/S IPV	Half of the women described threats, depravation, and displacement as a result of the guerrillas and paramilitaries in Colombia and identified that violence as the reason they moved to Ecuador.
**Social isolation**		
Restricted mobility	Only 19% of the sample of Colombian women reported they could leave the city without their partner’s permission. 14.7% reported that their partners tried to limit their contact with their family, which was correlated with P/S IPV (*p* < 0.001) in bivariate but not multivariate analysis. There is not quantitative information about immigration status.	Half of the women were prohibited by their husbands from leaving the home or engaging in community social life. The women were prevented from having friends, and their husbands use guilt, public humiliation, and physical force to keep them contained in the home. Several women explained how their immigration status prevented them from crossing the border into Colombia to visit family.
Lack of social support	Forty percent of Colombian women in the sample reported having family in the area when they moved to the neighbourhood, and on average had 1.7 family members living in the area. Having family in the area was negatively associated with emotional IPV (AOR: 0.50, 95% CI: 0.26, 0.96; *p* < 0.05). 37.3% reported disagreeing or somewhat disagreeing with the statement “I can count on I can count of my neighbour to watch after my house; 12% with “I feel part of the community”; and 62% with “I feel alone”. These were not associated with IPV outcomes.	A majority of the women had limited contact with friends and family in Colombia, and very few had relatives in Ecuador. All of the women denied having close, trusted friendships in their new communities.
Anti-Colombian sentiment	Over 30% of Colombian women reported that someone in their household experienced discrimination in the last six months because of their country of origin; 6 % reported discrimination because of race/ethnicity. These were associated with IPV in bivariate but not multivariate analysis.	Several women described experiences of discrimination in securing employment or access to social services. They also described a sense of separation and sometimes fear of their neighbours.
**Economic stress**		
Financial instability	75% of sample rents their home. In bivariate analysis only, household wealth was borderline significantly associated with P/S IPV (*p* < 0.11). Renting and food consumption score were not associated with IPV in bivariate analysis, but renting was a risk factor for P/S IPV in multivariate analysis.	All of the women faced conditions of poverty that generated marital stress and negatively impacted their abilities to integrate and succeed in Ecuador. Sources of income were unreliable so there was no ability to consistently pay bills or buy food. All but four women rented their homes. Of the four who did not rent, none had legal ownership of their properties. All of the women described facing food insecurity at some point, relying on diets of rice and potatoes, with no neighbours to turn to for support.
Underemployment	95% of male partners were employed at baseline and 42.7% of women were employed. Gaining employment for women was positively associated with P/S IPV in bivariate analysis (*p* < 0.05) and borderline positively associated in multivariate analysis (AOR: 3.1; 95% CI: 0.94, 10.13; *p* = 0.06).	Among the men, unemployment was common due to limited job opportunities, disability, illiteracy, or alcoholism. Among those who did work, wages were irregular and insufficient. The women faced underemployment because of housekeeping and childcare obligations, the low value placed on female labour, and gendered expectations that confined her to the home.
Financial dependence	Value of female-owned assets was negatively associated with P/S IPV in multivariate analysis, although it was of small magnitude (AOR: 0.998; 95% CI: 0.995, 1.0; *p* = 0.02). Male-to-female ratio of ownerships of household goods (value) and self-reported earning difference were not associated with IPV.	Even among women who worked part-time and earn their own wages, all but one of the women relied mainly on their husband’s income for survival and described limited ownership of household resources. Several women connected their financial dependence with the inability to leave their abusive husbands.

### Living on the border and legal status

Though surveys did not ask women about their legal status, many women who were interviewed discussed their lack of formal documentation. They described the challenges they faced trying to integrate into Ecuadorian society without the necessary visas, the stress of their immigration status seemingly underpinning all their social and economic interactions. For instance, when asked if she had any questions before beginning the interview, Valeria immediately asked:

*“Those of us who they won’t give papers, what can we do so that they give us the visa?” (Valeria, age 40, Nueva Loja)*

Lack of appropriate documentation meant that women feared that they would be denied re-entry into Ecuador if they crossed the border back into Colombia. When asked by the interviewer why she only contacted her family by telephone, Maria explained:

*“Because they have prohibited us from traveling there. We can go as far as Tulcán… because they have given us the visa and they said to us that if we pass through [the border], they will take away our visas. So we cannot go to Colombia.” (Maria, age 30, Tulcán)*

Sonia expressed a longing to return to her father in Colombia, but was unable to afford passports for her children, who were eligible for Ecuadorian citizenship:

*“Because of the Ecuadorian girl [daughter with citizenship], I can have Ecuadorian nationality… The problem is that there wasn’t money for the passports. They are 25 dollars each and we are three people. That’s 75 dollars, and where is that?” (Sonia, age 28, Tulcán)*

Another characteristic of the border region was the porous economy. Rosa (age 25, Tulcán) explained that in the absence of formal employment, her husband earned money by smuggling gasoline over the border. Similarly, after losing his job as a teacher, Esther’s (age 40, Nueva Loja) husband generated income by crossing the border, buying clothing in Colombia, and selling them for profit in Sucumbíos. In both these cases, smuggling was portrayed as a last resort after facing difficulties securing employment in the formal sector, which involved a safety and legal risk. Participant reports highlighted how the border region’s lack of an accountable police presence and the emergence of interstate crime rings not only allowed for such smuggling activities, but also facilitated human trafficking. Anita from Carchi described how her husband kidnapped and sold her son from a previous marriage:

*“My son of seven years old, he saw [my] husband with his girlfriend in the act [having an affair]. Being that he saw them, they took my son to sell him.” (Anita, age 47, Tulcán)*

Her son was sold to a wealthy family of cattle farmers, and Anita had to sell all her possessions and livestock to buy him back. She indicated that this was not an uncommon experience for families in this region.

### Violence along the continuum

Critical to their experiences of IPV in Ecuador were the women’s personal testimonies of trauma and violence prior to displacement. Approximately half of women interviewed pointed to guerrilla violence in their hometowns of Colombia as motivation for moving to Ecuador:

*“We came here because we were threatened by the paramilitaries and the guerrillas. They would pass through… some would make a problem for us, and [it was] better that we leave.” (Maria, age 30, Tulcán)*

Carolina from Sucumbíos described the decision to leave after guerrilla forces took control of her home and community:

*“There we couldn’t eat anything, feed ourselves anything, they arrived and occupied the kitchen, occupied everything.” (Carolina, age 40, Nueva Loja)*

These results were supported by our quantitative analysis that showed that over 40% of Colombian women who migrated to the area in the last 20 years, were forcibly displaced, meaning they moved because of fear of violence, political reasons, food insecurity, property destruction or other reasons not related to marriage or looking for work (see Table 2). Forced displacement was associated with five times the odds of experiencing physical and/or sexual violence (AOR: 5.06; 95% CI: 2.03, 12.59; *p* < 0.001; see Table 4). Migration-related adversity was also associated with IPV—such as having someone in the household verbally threatened (AOR: 4.37; 95% CI: 1.65, 11.60; *p* < 0.001; see Table 4) or wounded/physically attacked (AOR: 4.62, 95% CI: 1.25, 17.06; *p* < 0.05; see Table 4).

Several women in the qualitative sample also described childhood physical and sexual trauma. Pre-migration, Renata (age 42, Tulcán) was abused and abandoned by her parents at a young age and then raised by a neighbour. Her experience with IPV later in life included coerced sex and severe physical assault from her husband. Similarly Sonia, who was raised by domestic employees and felt abandoned by her parents, shared a story of child sexual abuse in Colombia:

*“The employees abused me from the age of five, I was raped at five years old. My father and mother never realised. I thought that one’s body was like a toy, I thought I wasn’t worth anything.” (Sonia, age 28, Tulcán)*

She became pregnant at 17 and described some of the most severe experiences of physical and emotional IPV in the qualitative sample. Her story spoke to the continuity of trauma and abuse. In most of these cases, whether it was guerrilla violence or domestic abuse, the trauma contributed to their decision to migrate. Unfortunately no quantitative data was collected on childhood maltreatment so we could not triangulate these results.

Post-migration, qualitative and quantitative data showed evidence of IPV in various forms (see Table 5). The women in the 15 interviews described a variety of experiences with IPV ranging from verbal abuse to physical and sexual violence. Several women described public humiliation or verbal abuse in the form of threats or name-calling. Other women described physical violence that included pushing, punching, kicking, and hair pulling. Paula (age 33, Nueva Loja) was hospitalised from a broken tailbone, Sonia (age 28, Tulcán) from a blow to the head when she was seven months pregnant. Renata’s (age 42, Tulcán) husband split her lip and caused her to need stitches. Renata and Alegria (age 45, Tulcán) both reported calling the police on their husbands for physical violence in the past. Some women reported sexual violence through manipulation and physical force. Most credited financial stress or jealousy as the cause of marital conflict. IPV for some was a daily occurrence, and for others was random and unprovoked. Overall, the abuse described in the interviews was continuous and isolating. Similarly, quantitative data found that IPV at time 1 was highly correlated with IPV at time 2 (see Table 3).

### Social isolation

#### Loss of support networks

Women in the interviews had limited contact with their friends and family in Colombia which minimised their ability to cope with IPV. A few women had a relative living nearby; many saw family once or twice a year or were limited to telephone correspondence. Several women reported having no contact at all. Forty percent of Colombian women in the quantitative sample reported having family in the area when they moved to the neighbourhood, and on average had 1.7 family members living in the area (see Table 2). Having family in the area was associated with lower risk of experiencing emotional IPV (AOR: 0.50; 95% CI: 0.26, 0.96; *p* < 0.05; see Table 4) but there was no statistically significant association for physical and/or sexual IPV (AOR: 0.66; 95% CI: 0.26, 1.64).

Not only did women lose their social support networks when migrating out of Colombia, but many were unable to develop such a network in their new Ecuadorian communities. Quantitatively, 37.3% reported disagreeing or somewhat disagreeing with the statement “I can count on neighbour to watch after my house”; only 12% agreed with “I feel part of the community”; and 62% said “I feel alone” (see Table 2). None of the women in the qualitative sample reported having close, trusted friendships in their new communities, not even Emilia (age 44, Tulcán), who had lived in Ecuador for 40 years. Alegria (age 45) had lived in Tulcán for 14 years but had no contact with family over the last 10 years. She had no friends in the community, and never left the house out of fear of being laughed at for a physical disability. When asked by the interviewer why she never became accustomed to life in Ecuador, Rosa explained:

*“The people there are different than the people here. Besides the violence, the people [in Colombia] for example, I go and I say, ‘look neighbour, I don’t have [something]…’ and I ask her and she lends it to me, whereas here you see their faces but don’t stop to ask... It’s something that gives you fear. You go to the health centre [and] there are people that only look at you, it deflates you and I don’t like it.” (Rosa, age 25, Tulcán)*

Participants reported challenges engaging socially with their new neighbours, which often seemed to stem from real or perceived anti-Colombian sentiment. Over 30% of women in the quantitative sample reported that someone in their household experienced discrimination in the last six months because of their country of origin (see Table 2).

#### Restricted mobility

Though much of this social isolation was situational, brought on by migrating away from friends and family into a new and different context, a lot of it was also intentional. Throughout the interviews, one of the prevailing themes was the confinement of women to the household and restricted socialisation imposed upon them by their partners. Over 80% of the quantitative sample of Colombian women reported they could not leave the city without their partner’s permission, and 14.7% reported that their partners tried to limit their contact with their family (see Table 2).

Women in the qualitative sample described partners who used guilt, shame, and physical force to keep their wives at home; the women were suspected of gossip, child neglect, or infidelity for spending any length of time away. Sonia (age 28, Tulcán), Maria (age 30, Tulcán), and Valeria (age 40, Nueva Loja) all said their husbands disliked them leaving the house and would become suspicious or accusatory if they went out. Esther described the same pattern:

*“He doesn’t like that I go out alone… When I have to go out, I have to go out with him, so I [stay] here. Like I told you, I rarely go out to run an errand or for something urgent, we always go out together… I don’t know if it’s because he doesn’t want to stay home alone, I don’t know, he doesn’t like that I go out and he doesn’t let me go.” (Esther, age 40, Nueva Loja)*

Renata explained that she preferred to stay home to avoid public humiliation from her verbally abusive husband:

*“I return to the house because it embarrasses me when other people hear everything that he says to me.” (Renata, age 42, Tulcán)*

Several of the women felt the effects of their isolation particularly strongly, to the point where they reported feeling trapped. Anita (age 47, Tulcán) described her sense of isolation as “feeling like a cell phone turned off” as if she was completely secluded from the outside world. During a jealousy-induced altercation, Esther was physically constrained to the house:

*“He hit me, he locked the doors so that I couldn’t leave.” (Esther, age 40, Nueva Loja)*

This isolation and lack of social support disempowered women against abusive husbands. Renata spoke about a time she went to the local women’s shelter after a particularly violent domestic dispute. Her husband was sent to a health clinic for alcoholism but escaped after a month and a half and returned home, and when the abuse continued, the police, doctors, and lawyers were unable to help her. Asked by the interviewer if she had ever tried to leave the relationship, she said:

*“It makes me afraid. He is a scary man.” (Renata, age 42, Tulcán)*

### Economic stress

#### Financial instability and underemployment

Much of the domestic violence experienced by Colombian women in this sample can be quantitatively and qualitatively associated with economic stress. Quantitatively, 95% of the sample reported that their male partners were engaged in the labour force, compared to just 52% of women. However, qualitative data showed that employment was often informal and irregular, and that even among working husbands, income was insufficient and inconsistent.

*“The little amount that they pay him is not enough for the rent.” (Carolina, age 40, Nueva Loja)*

Several men faced abrupt termination of employment or wage theft. Esther’s (age 40, Nueva Loja) husband worked as a schoolteacher and his wages were withheld for a month, which put a lot of financial strain on the family. Paula’s (age 33, Nueva Loja) husband was fired unexpectedly when he was accused of stealing a car. Rosa’s (age 25, Tulcán) husband lost his fingers in a work-related accident and was fired without pay. Others could not work due to disability, illiteracy, or alcoholism.

Women also described difficulties with underemployment. Valeria (age 40, Nueva Loja) and Isabel (age 47, Tulcán) attributed their unemployment to their husbands who forbade them from leaving the house to work. Quantitively, women joining the labour force was marginally associated with higher risk of physical and/or sexual violence (AOR: 3.09; 95% CI: 0.94, 10.13; *p* = 0.06; see Table 4).

Lack of legal status was another factor preventing both Colombian men and women from securing consistent, well-paid employment:

*“I am denied the documents, no one will employ me… I go out to look for work and they don’t give me it, they say no because I don’t have documents.” (Claudia, age 31, Nueva Loja)*

Facing these conditions of underemployment and financial instability, couples had to negotiate how to spend their limited funds, whether on rent, food, school fees, or leisure, and many women pointed to this as the source of fights and agitation:

*“When you don’t have anything to throw in the pot, you feel agitated and throw blame at your spouse… However, if you have food or something to cook, you change because at least you have enough to cook and there are no fights.” (Valeria, age 40, Nueva Loja)*

For many women, the stress of unemployment and food insecurity created friction and fighting, especially when couples disagreed about how to spend their money. Paula (age 33, Nueva Loja), who was often looking for part-time work to supplement her husband’s income as a delivery driver, described her husband’s pattern of spending their limited resources on drinking and socialising:

*“I strive to find some little job to help him with the expenses in the house and he, what he does is go out to play with his friends… [I told him], ‘Leave to go drink, it’s the only thing you know how to do. Lately you don’t give a shit about your kids. Doesn’t it embarrass you to see your daughter without shoes?’” (Paula, age 33, Nueva Loja)*

This particular fight ended with Paula hospitalised for a broken tailbone.

#### Housing insecurity

Mixed methods data (Table 5) also showed how housing insecurity contributed to marital stress and IPV. Quantitively only 25% of the sample owned their home (see Table 2), and renting (compared to owning) was a significant risk factor for physical and/or sexual IPV (AOR: 4.42; 95% CI: 1.95, 10.05; *p* < 0.001; see Table 4). Only three women in the qualitative sample owned their homes. Of the three, however, none of the houses were actually in the name of the woman. For Claudia (age 31, Nueva Loja) and Valeria (age 40, Nueva Loja), their husbands had sole ownership of the home, and both women mentioned in their interviews the ability of their husbands to throw them out of the house at any given moment. Though Karla lived in a family-owned home, documentation status prevented both her and her husband from officially owning the property themselves:

*“We still don’t have the Ecuadorian ID, not me or my husband. [The house] is in the name of [her daughter], of the little thirteen-year-old girl.” (Karla, age 52, Tulcán)*

Karla, who had lived in Ecuador for 26 years, was in the process of obtaining legal residency so that she could transfer the home out of her daughter’s name. However, she said she would have the house transferred to her husband’s name only, despite the fact that he had a severe head injury and could not easily move or speak, because he was considered “the boss”. Despite her husband’s disability, he often exerted his power over her through the use of verbal and physical violence.

“*He told me to fuck off when it was time to put him in bed, and he yanked my hair.” (Karla, age 52, Tulcán)*

Even though Karla was the main provider and caregiver for her family, the gender norm that men are the heads of the house prevailed over her right to home ownership. Due to her status as both woman and undocumented, Karla had no legal right to her own home.

The women who rented alluded to poor housing conditions and experienced difficulty making regular rent payments. Several women described borrowing money from relatives or neighbours to pay for rent or utilities. Isabel (age 47, Tulcán) was brought to tears during her interview remembering a time she was almost suddenly evicted.

#### Financial dependence

Financial dependence was a shared experience among the women of the qualitative sample, even among those who worked part-time and earned their own wages. In the quantitative sample, 85% of women reported earning less than their partners (see Table 2). However, self-reported earning difference were not associated with IPV (see Table 4).

In the qualitative sample, many felt like they did not have a right to or ownership of their household resources:

*“Other women when we go out somewhere, they work and they have their own wallets and have their money, but now I have nothing.” (Daniela, age 30, Tulcán)*

Daniela described a fight with her husband about the shop he operated out of their house. When he came home to find his wife selling milk to their neighbour, he publicly insulted her and forbid her from conducting business again.

Carolina, who occasionally earned money selling fruit on the street, told her husband she would leave him after an episode of physical violence, knowing that on her own she could not make enough money to survive.

*“If I live alone then I will work alone even though I won’t earn enough. ‘But I will not have the beating,’ I told him.” (Carolina, age 40, Nueva Loja)*

A lower earning potential and lack of control over financial resources acted as a barrier to safety for women experiencing IPV. Quantitatively, higher value of women-owned assets was negatively associated with physical and/or sexual IPV in multivariate analysis, although it was of small magnitude (AOR: 0.998; 95% CI: 0.995, 1.0; *p* = 0.02; see Table 4). However, male-to-female ratio of ownerships of household goods (value) was not associated with IPV in our quantitative sample.

An anecdote from the interview participants illustrates this connection. Valeria recalled a time when she tried to leave her marriage, but she had no money of her own and no right to their property. Their plot of land was in her husband’s name and she did not have enough money to leave:

*“I told him to stay with the property and that I would leave, I had already decided. Then at the last minute I decided, ‘ok, I will go but you give me a part of the money so that I can go,’ then he said no.” (Valeria, age 40, Nueva Loja)*

Without a claim to the land, a place to go, or money to leave with, she was forced to stay.

## Discussion

Our analysis revealed that for our sample of Colombian women living in the border with Ecuador what was supposed to be a transitionary period and transitionary place became the permanent context in which they experienced IPV. Migration is typically a temporary move, but displacement and long-term residence in the border region resulted in continuous physical, social, and economic insecurity, intensifying the conditions that facilitate IPV and limiting their ability to escape it. Our study highlights four main mechanisms by which displacement influenced the social and economic realities of these Colombian women years after crossing the border. First was the issue of legal residence and documentation. Even though Ecuador had one of the highest refugee recognition rates in Latin America at the time of data collection, granting asylum to 66% of applicants, displaced persons faced long wait times for short-term temporary visas [17]. Refugees were given renewable identification cards that expired every three months while waiting for an asylum interview, a wait that could take as long as two years [17]. If they were finally granted asylum, their refugee visa was valid for only one year before they had to reapply. Thousands of Colombians who had crossed the border were in constant pursuit of the right to stay in Ecuador, or else faced the risk of exploitation or deportation. A lack of a companion integration programme left refugees granted short-term legal status in the margins of Ecuadorian society. Most women in this cohort did not have the money, time, or literacy to pursue permanent legal status. Whether it was the ability to obtain regular, formal employment, the ability to own a home, or even the ability to cross into Colombia, their liminal immigration status created conditions through which IPV was able to continue or escalate.

Second, following previous research documenting that women previously exposed to violence are at increased risk for later revictimisation [28] we found that women in our sample experienced violence along life course and migratory continuums. Several women in the qualitative sample disclosed physical or sexual abuse either as children or in prior marriages, and many of the women described long-term IPV in their current relationships. Quantitatively, while no data were gathered on childhood maltreatment or IPV in previous relationships, IPV at time 1 was highly associated with IPV at time 2. Women also experienced a continuum of violence related to migration. Both qualitative and quantitative data showed how exposure to guerrilla violence and subsequent displacement contributed to the risk of IPV. The Refugee Council reports that Colombian migrants are for the most part unwilling to leave the border region for fear of intensified discrimination in central and southern Ecuador, while others remain to stay close to family in Colombia [17]. Remaining in the border region meant continued exposure to the guerrilla violence permeating from Colombia into Ecuador. As the presence of guerrilla and paramilitary groups intensified on the Ecuadorian side of the border, Colombian refugees were terrorised by the same violence that triggered their displacement, and liminal insecurity persisted. Exposure to conflict in their home country informed how men and women perceived violence and managed anger, which is a risk factor for IPV victimisation and perpetration, even after migrating away from the conflict [7, 9, 29]. In sum, the experience of migration and living in the border region facilitated IPV because security remained an ambiguous concept, inside and outside intimate relationships.

Third, our research found that the conditions of displacement and liminality generated social isolation which exaggerated the risk of IPV and limited the women’s ability to cope with it. Mixed methods results showed evidence that women in this study for the most part had few or no relatives nearby, had limited contact with family across the border, were severely restricted in their mobility by their partners, and faced difficulty developing social support networks in their new communities. Many interviewees spoke of an anti-Colombian sentiment that contributed to feelings of social alienation and prohibited refugee women from integrating into local schools, churches, and community groups, and likely from accessing IPV-related services such as law enforcement or women’s clinics. For those women with family nearby, survey results showed a protective effect against emotional violence only. The fact that no statistically significant associations were detected between physical and/or sexual violence and having family nearby could be explained by lack of statistical power, as less women in the survey reported physical and/or sexual IPV. Furthermore, it is important to note that the association found between having family members living in the area, and physical and sexual IPV trended in the same (protective) direction. Further qualitative exploration can help provide additional insight into why familial and social support may be more or less protective for specific types of intimate partner violence.

Interviews effectively revealed how strict gender roles aided men partners in restricting women mobility. The men used guilt, humiliation, and sometimes physical force, to accuse their partners of neglecting childcare and housekeeping duties and to confine women to the household. The men, who were commonly experiencing underemployment and marginalisation as migrants themselves, maintained a sense of power and masculinity by exerting control over the movement and social interactions of their woman partners. The inability to integrate socially into Ecuadorian communities suggests this dynamic was on-going and contributed to IPV. Our findings are in line with previous research documenting discrimination and adherence to traditional inequitable gender norms as risk factors for IPV [30–35], while social support acts as a protective factor [28]. This research highlights the deep interplay between these factors and how they are exacerbated by liminality.

Fourth, as has been documented in previous research [28], the insecurity of employment and income were identified as significant drivers of IPV in our study. Displaced men often face difficulties finding work in urban labour markets, where their agricultural skills and low levels of educational attainment have little value [36]. Displaced women, however, are more easily able to find work in the informal sector, predominantly in domestic work, as reflected by the number of Colombian women in this cohort working in laundry services. Hence, while quantitative results showed that the majority of men were participating in the labour force in some way, qualitative data revealed that those jobs were low-paying, irregular, and insecure, and that the stress and insecurity of poverty greatly contributed to IPV. Men’s inability to provide for their families financially threatens patriarchal constructions of masculinity which revolve mainly around the expectation of men as breadwinners [23, 37]. Men in this situation seek to restore the lost power in the household by using violence against their partners [36]. Conditions of displacement exacerbate IPV because when poverty necessitates female employment, expectations about the division of labour are disrupted and notions of masculinity are undermined; the indefinite liminality of the border region signifies a new reality for intimate partners that contradicts pre-existing ideas about economics and gender.

Interestingly, our quantitative results found that higher value of women-owned assets was protective against IPV (although of small magnitude) but entering the labour force for women was a risk factor. While migration could contribute to decreases in IPV by improving women’s access to resources [38]—which, in turn, could facilitate equalization of bargaining power within a relationship and increase women’s ability to leave abusive relationships—research among internally displaced women found that working to supplement their husband’s insufficient incomes was seen as untraditional and transgressive [36] in line with worldwide evidence that shows that in countries where few women work, working for cash increases a woman’s risk of IPV [33]. In our qualitative sample, women were either discouraged by their partners from working, too busy with childcare, or employed in the informal market in housekeeping or food service, which remains within the traditional women sphere. This could also be due to romantic jealousy, as men fear that women entering the work force might open possibilities for unfaithfulness [39–41].

There are some limitations to this study. As a secondary data analysis, the qualitative and quantitative data did not encompass all relevant questions or indicators for our research question. Further, sample size for the quantitative data was calculated for the original study whereas we used a sub-sample for our analysis. Selecting a sub-sample might have rendered the sample under-powered to detect associations with some of our secondary outcomes. However, concentrating on the experiences of IPV among Colombian refugees specifically allows for a more nuanced discussion on the pathways of IPV for a particularly vulnerable group. Finally, both qualitative and quantitative data may be subject to social desirability bias around this sensitive topic; however, WHO-recommended procedures for collecting IPV data were enacted to both reduce participant discomfort and protect their confidentiality and safety. The analysis also has several strengths, including pairing a strong theoretical foundation with robust empirical analysis. We triangulated quantitative and qualitative data which contributes to increasing the credibility and validity of our results. Our quantitative data came from longitudinal survey data, whereas most IPV research has examined correlational associations. This allows us to make more robust inferences and contributes to a significant research gap in the region—in a recent review of studies that prospectively identified predictors of IPV, none of the 60 studies were conducted in South America [42]. Finally, by employing a liminality framework, the study comprehensively considered a range of factors to understand the impact of displacement on the experience of IPV women.

### Implications for policy and programming

Our study emphasises the importance of keeping the safety of women who have migrated at the forefront of policymaking and resource distribution. Currently the region’s economy, housing and social infrastructure, and social cohesion have been deeply impacted by the Venezuelan migration crisis, as well as the COVID-19 pandemic [43]. Women in living in liminal existence will bear the brunt of this social upheaval. This research underscores the need for policy makers to consider how the long-term marginalisation of refugee and migrant women contributes to their victimisation. This research can also contribute to the International Organisation for Migration (IOM), United Nations High Commissioner for Refugees (UNHCR) and other international development agencies’ programming designed to improve the health and social outcomes among displaced people. These agencies aim to reduce displaced people’s vulnerability by supporting access to safe housing, employment, health care and education, and integration into receiving communities. Our findings support ensuring inclusion of all migrants in their programming, regardless of their documentation status, as well as incorporating IPV prevention and response programmes into their services.

Furthermore, interventions with gender-synchronised [44], gender transformative approaches [45, 46] that allow for a redefinition of traditional gender norms, such as wife-beating acceptability and the role of men as breadwinner, and which also tackle relational aspects in the couple, such as open communication and trust [47, 48], are desperately needed. These initiatives need to be paired with more structural initiatives that allow families to access resources and reduce poverty-induced tension and conflict.

## Conclusions

In summary, mixed methods analysis results highlighted the various ways in which the unique physical, social, and economic circumstances of displaced Colombian women contributed to IPV. We found qualitative and quantitative evidence that displaced women experienced violence on multiple continuums and that previous trauma makes women increasingly vulnerable to IPV. Both surveys and interviews revealed how common it was for male partners to control their wives’ movement and social interactions, and both sets of data displayed how food and housing insecurity and financial dependence perpetuated IPV. Furthermore we demonstrate how the uniquely liminal conditions of the border region expose women migrants to new forms of violence while also limiting their access to safety.

With lack of legal documentation underpinning every facet of their daily lives, and their marginal status as impoverished, women migrants were at an increased risk for IPV. Moving to Ecuador, Colombian women lost their social support networks and were unable to develop friendships or a sense of community in their new homes. Furthermore, lack of adequate employment opportunities, combined with a legal status that barred them from participating in the formal economy, or owning assets contributed to marital stress and locked Colombian families into an enduring state of financial vulnerability. Situated in the porous and insecure border region, and in the context of marriages with rigid gender roles, the Colombian women who experienced IPV lacked the financial and social resources to defend themselves or leave their partners.

## Data Availability

The datasets used for the quantitative analyses are publicly available on the Harvard Dataverse. References for these data sets are: Impact Evaluation of Cash, Food Vouchers, and Food Transfers among Colombian. Refugees and Poor Ecuadorians in Carchi and Sucumbíos: Baseline Survey [Internet]. Harvard Dataverse. 2016. Available from: 10.7910/DVN/YW4WIT. Impact Evaluation of Cash, Food Vouchers, and Food Transfers among Colombian. Refugees and Poor Ecuadorians in Carchi and Sucumbíos: Endline Survey [Internet]. Harvard Dataverse. 2016. Available from: 10.7910/DVN/AXGCHT. The qualitative dataset generated analysed during the current study are not publicly available due to research ethics board restrictions. However, summaries of the information are available from the corresponding author upon reasonable request. The interview guides for all study participants are also available upon request.

## Abbreviations

AOR: Adjusted odds ratio
CEPAR: Centro de Estudios de Población y Desarrollo Social
CI: Confidence interval
IFPRI: International Food Policy Research Institute
IOM: International Organisation for Migration
IPV: Intimate partner violence
LSHTM: London School of Hygiene and Tropical Medicine
UNHCR: United Nations High Commissioner for Refugees
US: United States
WFP: World Food Programme
WHO: World Health Organisation
